# Colonoscopy Practice in Lagos, Nigeria: A Report of an Audit

**DOI:** 10.1155/2013/798651

**Published:** 2013-02-27

**Authors:** C. A. Onyekwere, J. N. Odiagah, O. O. Ogunleye, C. Chibututu, O. A. Lesi

**Affiliations:** ^1^Gastroenterology Unit, Department of Medicine, Lagos State University College of Medicine/Teaching Hospital, P.O. Box 203 Satelitte Town, Lagos, Nigeria; ^2^Gastroenterology Unit, Department of Medicine, Lagos State University Teaching Hospital, PMB 21266, Ikeja, Lagos, Nigeria; ^3^Clinical Pharmacology Unit, Department of Medicine, Lagos State University College of Medicine/Teaching Hospital, PMB 21266, Ikeja, Lagos, Nigeria; ^4^Cornell Health Centre, Surulere, Lagos, Nigeria; ^5^Department of Medicine, University of Lagos College of Medicine/Teaching Hospital, Private Mail Bag 12003, Lagos, Nigeria

## Abstract

*Background*. Colonoscopy effectiveness depends on the quality of the examination. Community-based report of quality of colonoscopy practice in a developing country will help in determining standard and also serve as a stimulus for improvement in service. *Aim*. To review the quality of colonoscopy practice and document pattern of colonic disease including polyp detection rate in Lagos, Nigeria. *Method*. A protocol that captured the patients' demographics, indication, and some quality indices of colonoscopy was developed and sent to all the identified colonoscopy units in Lagos to complete for all procedures performed between January 2011 and June 2012. All data were collated and analyzed. The quality indices studied were compared with guideline standard. *Results*. Twelve colonoscopy centers were identified but only nine centers responded. The gastroenterologist/endoscopists were physicians (3) and surgeons (5). Six hundred and seven colonoscopy procedures were performed during this period (M : F = 333 : 179) while the sex was not disclosed in 95 subjects. The examination indications were lower GI bleeding (24.2%), altered bowel habits (9.2%), lower abdominal pain (9.1%), screening for CRC (4.3%) and unspecified (46.8%). Conscious sedation was generally used while bowel preparation (good in 81.4%) was done with low residue diet and stimulant laxatives. Caecal intubation rate was 81.2%. Common endoscopic findings were haemorrhoids (43.2%), polyps/masses (13.4%), diverticulosis (11.1%), and no abnormality (23.4%). Polyp was detected in 6.8% of cases. *Conclusion*. Colonoscopy utilization is low, and the quality of practice is suboptimal; although limited resources could partly explain this, however it is not clear if the low rate of polyp detection is due to missed lesions or low population incidence.

## 1. Introduction

Colorectal cancer (CRC) is a common malignancy especially in the Western countries and the third leading cause of cancer death [1]. Recent reports indicate that it is also not uncommon in sub-Saharan Africa including Nigeria [2]. Early detection of colorectal cancer (CRC) has been shown to improve disease outcome [3]. Colonoscopy is the gold standard for colorectal cancer (CRC) screening [4]. There is therefore the need to ensure proper conduct of this procedure. Colonoscopy effectiveness ultimately depends on many variables related to the quality of the examination, which tends to be widely variable in clinical practice [5]. Many quality indices have been studied to optimise endoscopy procedure from the perspective of both the patient and the physician, including caecal intubation rate, adenoma detection rate, withdrawal time and quality of colonoscopy reporting. The later emphasises amongst others the documentation of caecal landmark as well. One clinical relevance of this is the occurrence of interval CRC, a marker of poor quality colonoscopy. Interval CRC is defined as that which occurs within a specified period of time after a colonoscopy (usually 5 years) [6]. Most interval CRCs occur because adenomas or actual CRC is missed during a colonoscopy. Several guidelines have been developed to enhance the quality of colonoscopy including the American Society for Gastrointestinal Endoscopy guideline [7] and the position statement of the European Society for Gastrointestinal Endoscopy [8]. Three aspects of quality are addressed by the American Society for Gastrointestinal Endoscopy (ASGE) guideline [7]: (1) risk stratification before the procedure, (2) the procedure itself, and (3) the post procedure including complications and surveillance strategy.

Colonoscopy is highly underutilized and even where used is underreported in sub-Saharan Africa; an earlier report [9] had indicated low level of practice of CRC screening. The few African reports [10, 11] showed indications and findings in relatively small study population with a number of them revealing advanced presentation of most cases of CRC in line with observed pattern in regions of low CRC screening [12, 13]. Also a report [14] of colonoscopy outcome from a tertiary hospital in Jos in Northern Nigeria showed polyp detection rate of 6%, a figure much less than that from Western countries. Most western reports indicate that a much higher rate and inability to detect adenomatous polyps in a minimum of 25% in men and 15% in females above fifty years of age during screening colonoscopy is adjudged to be poor quality performance [7]. But no report exists about compliance with quality indices amongst those performing colonoscopy in Nigeria sub-Saharan Africa. There is therefore a need to document colonoscopy outcomes in our population including our polyp detection rate which could serve as a quality indicator for future assessment of colonoscopy performance. The society of gastroenterology and hepatology in Nigeria is made up of health professional drawn from several disciplines whose interest is to foster the course of gastroenterology and is affiliated to the World Gastroenterology Organisation. This report will also serve to sensitize gastroenterology practitioners on need to improve on colonoscopy performance.

## 2. Aim

The aim of this paper is to review the quality of practice of colonoscopy in Lagos, Nigeria, and document our pattern of colonic disease including polyp detection rate.

## 3. Method

 This was a retrospective study and complied with all ethical protocols as contained in the Helsinki declaration. All patients undergoing colonoscopy were meant to sign written consent. A protocol that captured the following quality indicators (quality/completeness of reporting, bowel preparation, caecal intubation, polyp detection, and complication) was developed and used to ascertain the extent of compliance in various colonoscopy units in Lagos between January 2011 and June 2012. Also the demographics, indications, and the colonoscopy findings of the patients were to be recorded. A total of 12 colonoscopy centres were identified and contacted on phone about the survey. The protocol was sent to them to complete and return. They were either to record data of all patients seen in a centre during the study period in a form of cumulative data spread sheet or to fill out each patients' information on a form as they deem more convenient. Further calls and visits were made to them to clarify any issues about the protocols and to retrieve the completed forms from them physically where they were unable to send data by electronic transfer.

The bowel preparation was graded as good, fair, or poor as reported by the performing colonoscopist in line with the ASGE guideline in which good refers to a clear fluid in the colon, and fair refers to small residual semisolid faeces. According to the guideline the minimum caecal intubation rate is 90 for all colonoscopy procedures while the minimum recommended adenoma detection rate is 25 for men and 15 for women above 50 years. All data were collated and analysed using SPSS statistical package. Subjects' age distribution and quality of bowel preparation were presented as chart while colonoscopy indication and findings were presented in frequency tables. The quality indices studied were compared with guideline standard.

## 4. Results

A total of 12 colonoscopy centres were identified but we were only able to evaluate nine centres (one public and eight private) that responded. Eight centres returned in a form of cumulative data sheet while one had individual forms for each patient. Three (3) of the endoscopists were gastroenterology physicians, and five (5) were general surgeons.

The data of one centre (which had 152 cases) could not be analysed because of the form in which the data were submitted and so was not included in this report.

Six hundred and seven colonoscopy procedures were performed during this period (333 males, 179 females while the sex was not disclosed in 95 subjects).

### 4.1. Subjects Demographics

**Figure 1:**
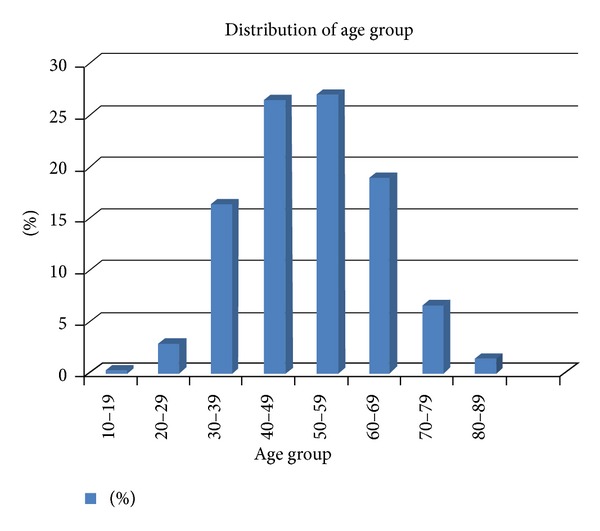
Age distribution according to decades of subjects undergoing colonoscopy.

Patients were aged between 17 and 89 years (mean:51.21 years); however the age was unreported in 42.7% of cases. Of 348 cases that had their ages reported, 54% were more than or equal to 50 years while the median age group was from 50–59 years. Fifty-five percent of them were males, while 29.5% were females; sex was unreported in 15.7% of cases (Figure 1).

### 4.2. Indications for Colonoscopy

The indications for colonoscopy examination are shown in Table 1. The commonest indications were lower gastrointestinal bleeding (24.2%), altered bowel habits (9.2%), lower abdominal pain (9.1%), screening for colorectal cancer (4.3%), unspecified (46.8%).

### 4.3. Premedication and Bowel Preparation

**Figure 2:**
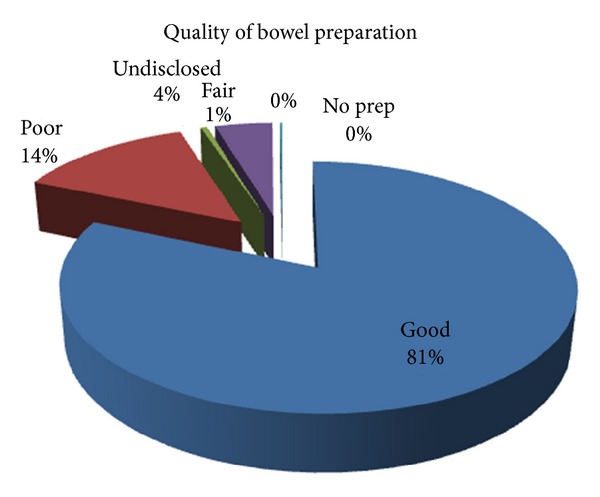


Premedication involved use of benzodiazepines, opioid analgesics, and antispasmodics. Bowel preparation for the subjects that had colonoscopy consisted of ingestion of only low residue diet for some days prior to examination and use of stimulant laxatives which included Epsom salt, Castor oil, and Picolax prior to examination. The quality of bowel preparation graded as from good to poor is shown in Figure 2 with good bowel preparation in 81% of them.

### 4.4. Caecal Intubation Rate

The caecal intubation rate 81.2% (the proportion of patients in whom there was insertion of the colonoscopy tip into the caecal caput) reflects a complete examination; permitting examination of the medial aspect of the caecum proximal to the ileocaecal valve is shown in Table 2.

### 4.5. Colonoscopy Findings

The findings are as shown in Table 3. The commonest findings were vascular lesions comprising mainly haemorrhoids (43.2%), polyps/masses (13.4%), diverticulosis (11.1%), and no abnormality (23.4%). The polyp detection rate was 6.8% (40). Of these 40, 25 were males, 11 females, while sex undisclosed in 11. Two-thirds of those having polyps were 50 years and above while the remaining third were less than 50 years old. Commonly affected locations with lesions were the anus (37.7%), rectum (3%), and sigmoid colon (3%).

### 4.6. Complications of Procedure

No complications were reported.

## 5. Discussion

Our data over an eighteen month period appear low when compared with reports [5, 15] from the developed countries including the Western nations. Chen et al. had analysed over 10 000 subjects in his assessment of the role of the endoscopist versus age and sex in predicting adenoma detection at colonoscopy examination. Similarly Baxter et al. studied the relationship between endoscopist characteristics determined from administrative data and occurrence of interval CRC in over 14 000 patients undergoing colonoscopy during a five-year period. The present finding is a reflection of low level of colonoscopy practice as had been reported earlier [10, 11]. High cost of colonoscopy services, low infrastructure, and awareness amongst other factors may be partly responsible. The scope of health insurance coverage is low and generally most people pay for their health bills (colonoscopy inclusive) off pocket.

One observation here is the preponderance of private health establishment in the study. There are two sides to healthcare facilities in Lagos; the government owned and the private sector led. The government hospitals have three levels of care: primary, secondary, and tertiary. There are two tertiary hospitals in Lagos, one of which participated in this study while the second is just commencing colonoscopy service. The other government hospitals at other levels of care do not offer colonoscopy services; hence the option available to those requiring the service is the private sector hospitals. However no difference is observed in the data between the solitary government hospital and the private ones.

Also there is an evident low level of colonoscopy reporting amongst the practitioners with a lot of the variables including age and sex not recorded. Lack of appropriate software for reporting colonoscopy may contribute to this. There is a slight male predominance amongst the study subjects and the indications for examination though similar to earlier report differed in more cases of rectal bleeding in this report [9, 14]. There is a low rate of CRC screening using colonoscopy in our environment, even though the mean age of the majority of subjects was over fifty years. This could contribute to late presentation of most cases of CRC as previous reports [12, 16] had indicated.

 Bowel preparation relied on having the subjects on low residue diet as well as use of stimulant purgatives which are known to cause abdominal discomfort including cramping and bloating from gas which could affect patience tolerance of the procedure and ultimately affect completeness of the examination. Although generally the quality of preparation was fair, this could be better with the use of more modern proprietary formulations using polyethylene glycol.

The caecal intubation rate (82%) is slightly below the recommended ASGE average. Although reasons for incomplete examination were not investigated, this could be related to patients' tolerance and calmness during examination which may be influenced by the type of bowel preparation and premedication as earlier reported. Brahmania et al. [17] in their study of maximizing completion rate of incomplete colonoscopy amongst gastroenterologist in a Vancouver Hospital noted reasons for incomplete colonoscopy to include; poor bowel preparation, pain or inadequate sedation, structural anomaly including tortuous colon, diverticular disease, and obstructing mass lesion. Most of the examinations in the present study were done using conscious sedatives as opposed to use of deep sedation with agents like propofol in developed countries. This fact had been alluded to by Nwokediuko and Obienu in a recent report [18]. It may also be a reflection of the level of expertise of the examiners. The low volume of colonoscopy performed may ultimately affect the level of skill and expertise as has been previously reported [19, 20].

 The colonic pathology observed is similar to earlier reports from Africa [9, 14] including the polyp detection rate (6.8%) which is much lower than that in Western literature. This may reflect the population incidence or an underestimation due to missed lesion resulting from inadequate bowel preparation and lower caecal intubation rate. Polyp prevalence has been reported to vary with age and sex as our report shows a male predominance and older age (>50 years), while some reports have noted geographical differences in polyp detection though such differences were ascribed to environmental factors including diet rather than race [21]. There are no published population-based data on the prevalence of polyp in our setting, although an earlier study [22] had noted a higher polyp detection rates in the white population. Also low incidence of colon cancer reported in our population has been attributed to a number of factors including dietary and rarity of predisposing colonic lesion polyps inclusive [2]. Further studies are required to validate this low polyp detection rate.

We were limited in not having histological data to confirm the nature of the observed colonic pathology and so unable to further classify the polyps and comment on the adenoma detection rate. The risks of the patients for CRC as well as withdrawal time during colonoscopy performance were not equally assessed as these have been shown to influence colonoscopy outcome.

Overall our colonoscopy practice is yet to attain the standard set in Western guideline; similar findings or worse (caecal intubation rate between 56 and 76%) were recorded in UK [23] over a decade ago but this improved with better training and infrastructure. It is our believe that this type of audit is going to be a regular exercise by the Society of Gastroenterology and Hepatology in Nigeria (Lagos chapter) to encourage an improvement in our endoscopy service.

## 6. Conclusion

There are too few colonoscopy centres in Lagos, with low volume of procedures and generally poor reporting of procedures. There is a low rate of CRC screening using colonoscopy in our environment. The observed caecal intubation rate is less than 90% recommended by most guidelines. There is also a low rate of polyp detection, when compared to international standards (US). It is not clear whether this is due to missed lesions (as a result of low rates of good bowel preparation and low caecal intubation rates) or due to a low incidence of polyps in our setting, as no prior population-based data are available.

## 7. Limitations of the Study

This retrospective analysis relied on data submitted by the endoscopists, so one cannot rule out underreporting. Our sample size appears small but this is a reflection of the level of practice of colonoscopy in our setting. 

The data of each colonoscopist were not individually assessed, but a general analysis was done to assess the overall reporting, performance and, outcome of colonoscopy in Lagos, Nigeria.

## Figures and Tables

**Table 1:** Clinical indications for colonoscopy amongst those disclosed.

Clinical indication	Frequency	Percentage
Lower GI bleeding	141	23.2
Abdominal pain	55	9.1
Colorectal cancer screening	26	4.3
Constipation	24	4.0
Others	18	3.0
Diarrhoea	13	2.1
Altered bowel habits	13	2.1
Anal pain	11	1.8
Anaemia	6	1.0
History of polyps/masses	5	0.8
Metastatic liver disease	4	0.7
Haematochezia + diarrhea	3	0.5
Haematochezia + constipation	3	0.5
Epigastric pain	1	0.2

Total	284	100.0

Total valid number = 284.

**Table 2:** Ceacal intubation rate.

Ceacal intubation	Frequency	Percentage
Undisclosed	18	3
Yes	493	81.2
No	96	15.8

Total	607	100

**Table:** (a)

Types of endoscopic findings	Frequency	%
Normal	55	9.1
DD and vascular	142	23.4
Ulcerative and vascular	4	0.7
Mass and colitis	3	0.5
Colitis and vascular	4	0.7
Melanosis coli and haemorrhoids	2	0.3
Megacolon	3	0.5
Upper GI	5	0.8
Adhesion/obstruction	2	0.3
Extrinsic compression	1	0.2
Perianal abscess	1	0.2
Ulcerative lesion	8	1.3
No colonic haustrations	1	0.2
Natal cleft intertrigo	1	0.2
Anal cushions	1	0.2
Long sigmoid	1	0.2
DD and ulcerations	1	0.2
Spastic colon	1	0.2
Mass/inflammation/haemorrhoids	3	0.5
Mass/DD/haemorrhoids	1	0.2
Ulceration and colitis	1	0.2
Masses/growth	57	9.4
Diverticulosis	39	6.4
Erosive colitis	18	3.0
Vascular lesions/haemorrhoids	213	35.1
Masses and diverticulosis	4	0.7
Masses and vascular	13	2.1

Total	607	100.0

*The polyp detection rate was 6.8%. DD: diverticular disease.

**Table:** (b)

Location	Frequency	%
None	255	42.0
Ceacum	3	0.5
Ascending colon	7	1.2
Transverse colon	6	1.0
Descending colon	5	0.8
Sigmoid	18	3.0
Rectum	18	3.0
Anus	229	37.7
Pancolitis	12	2.0
Multiple locations	54	8.9
